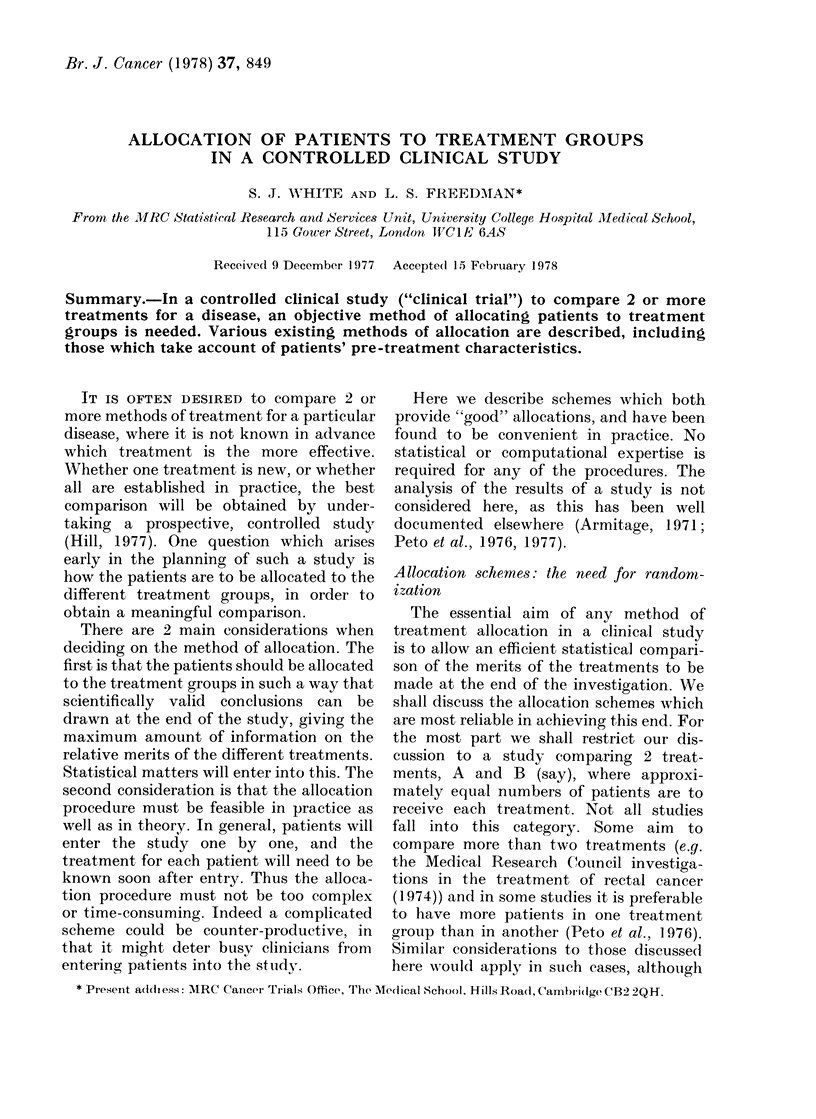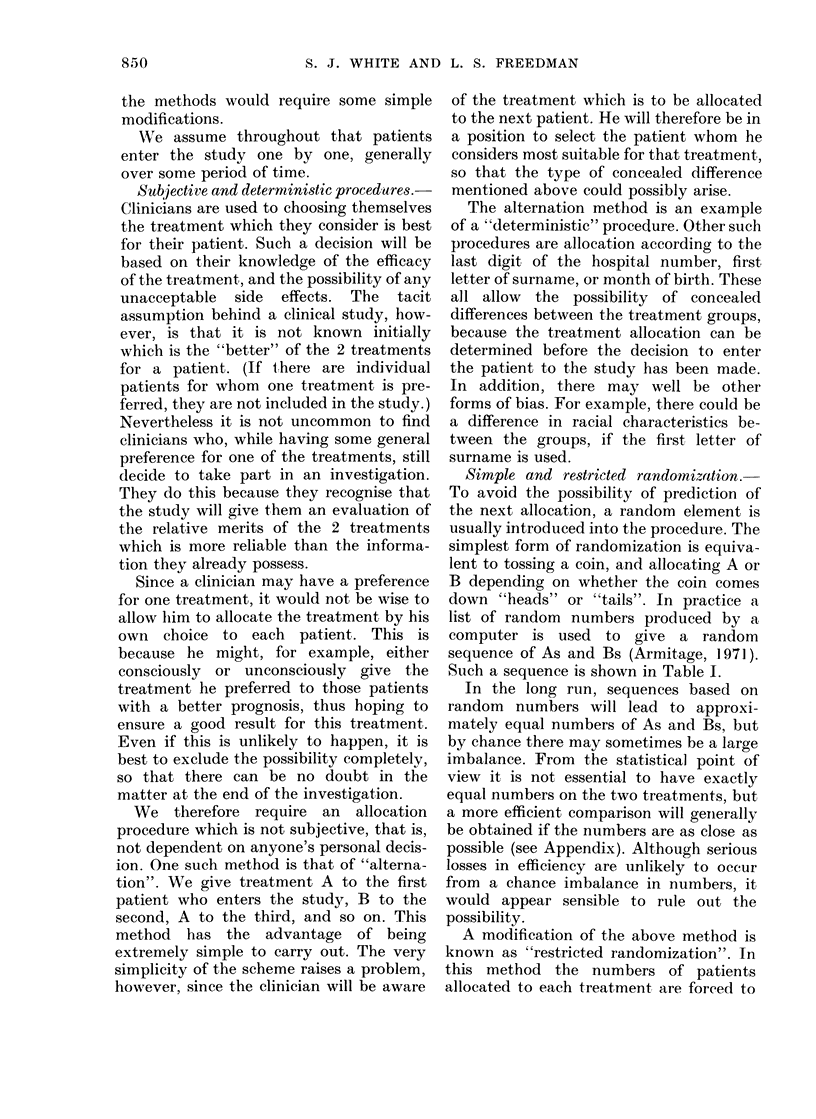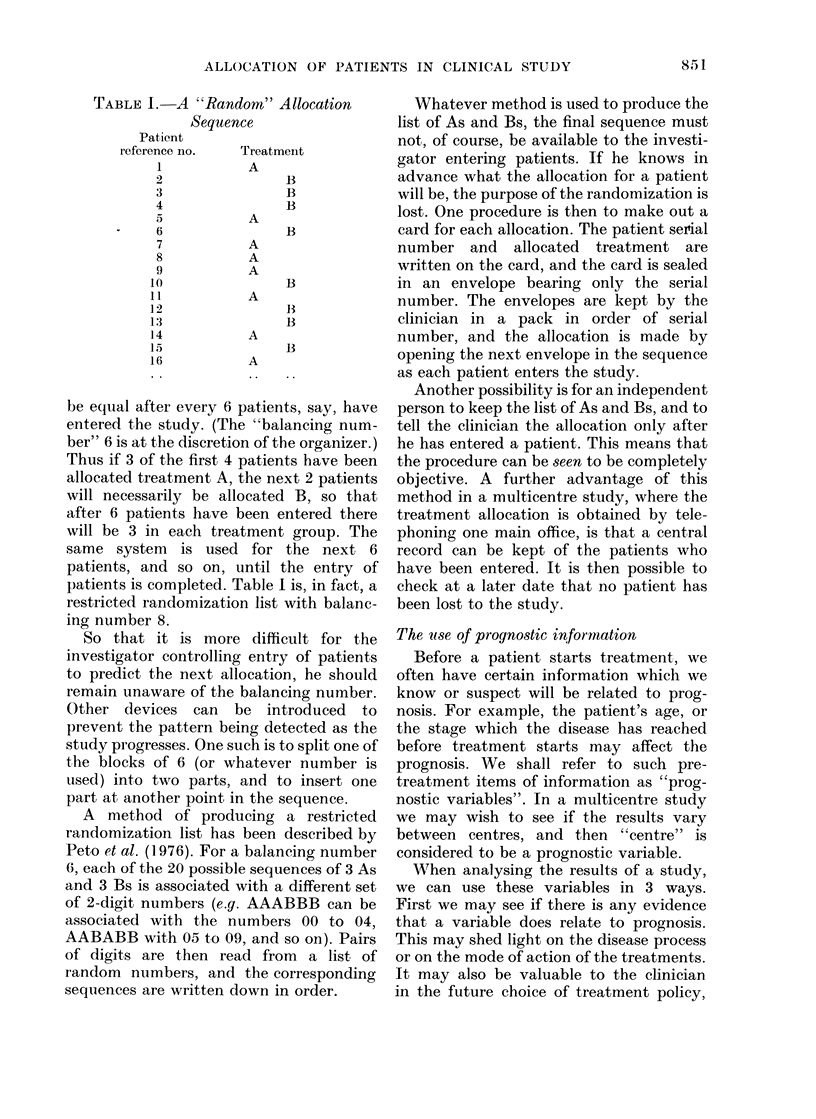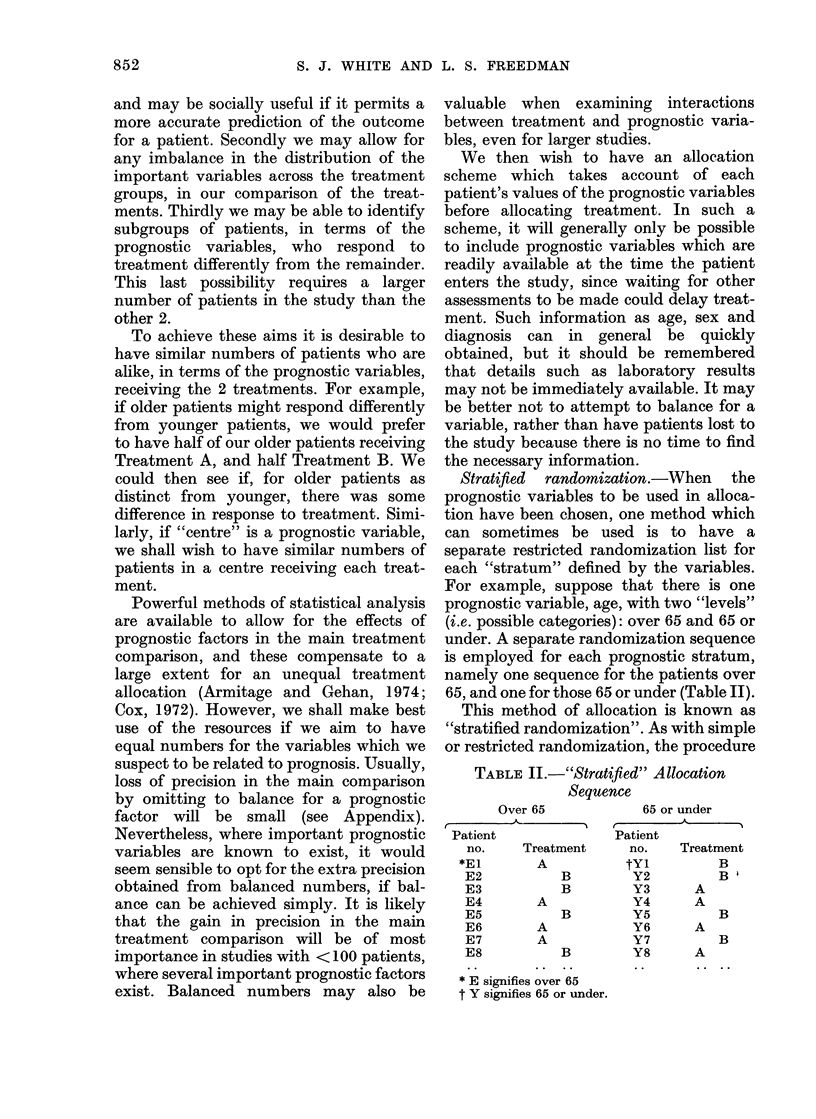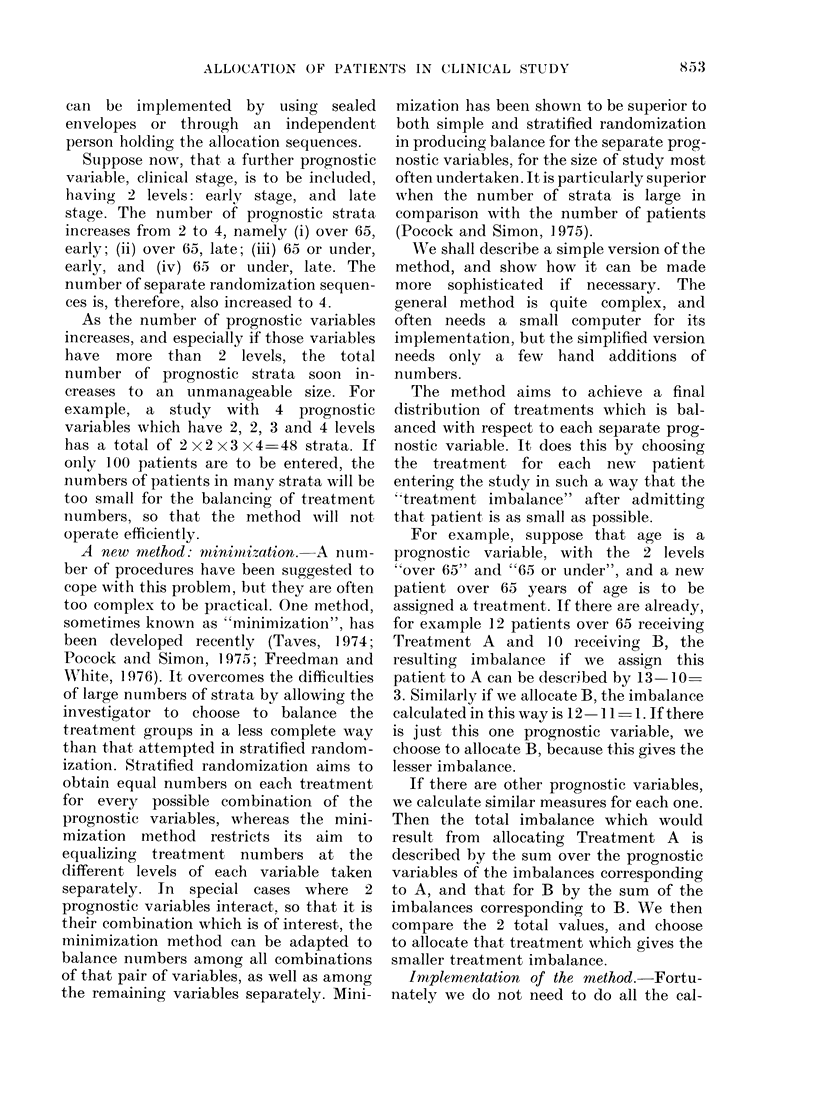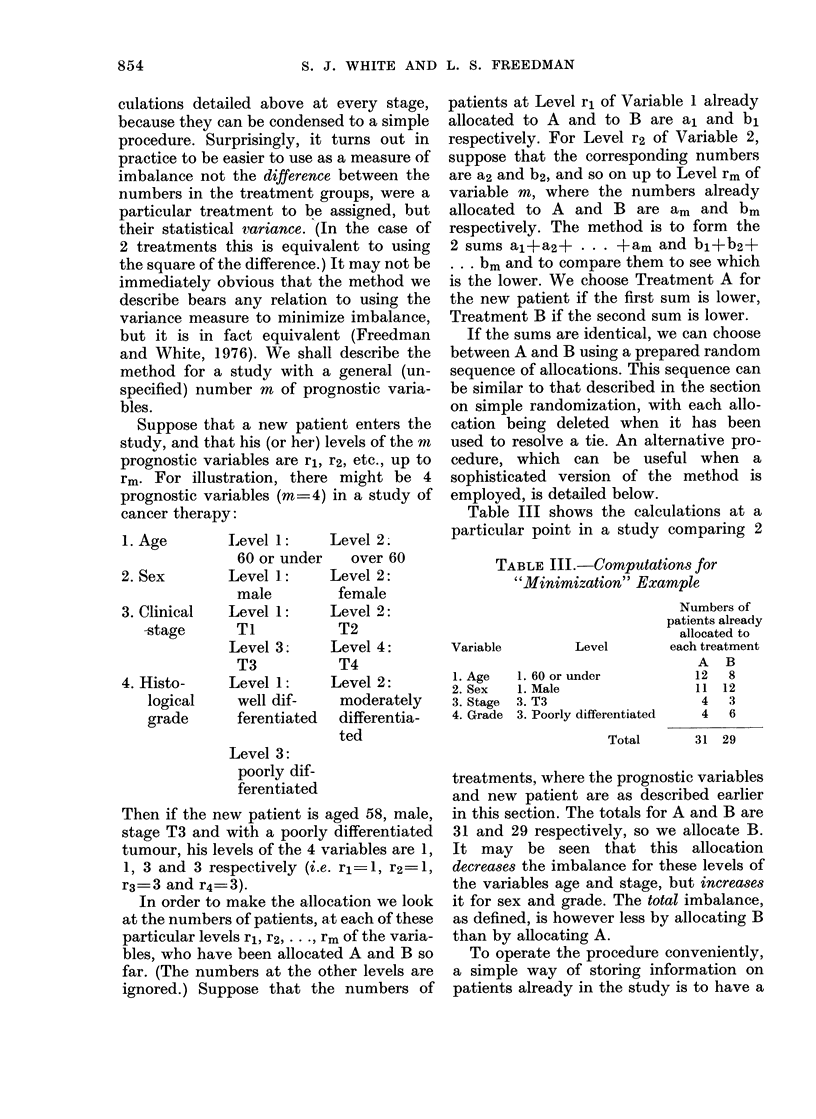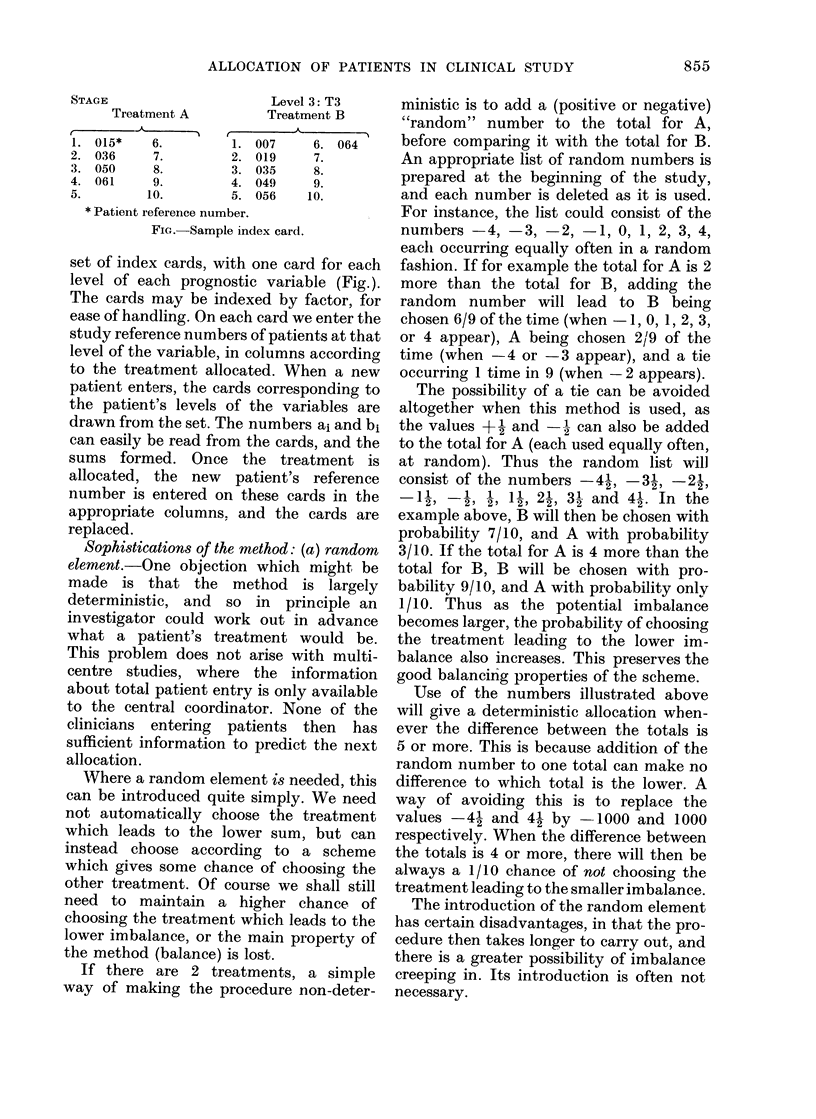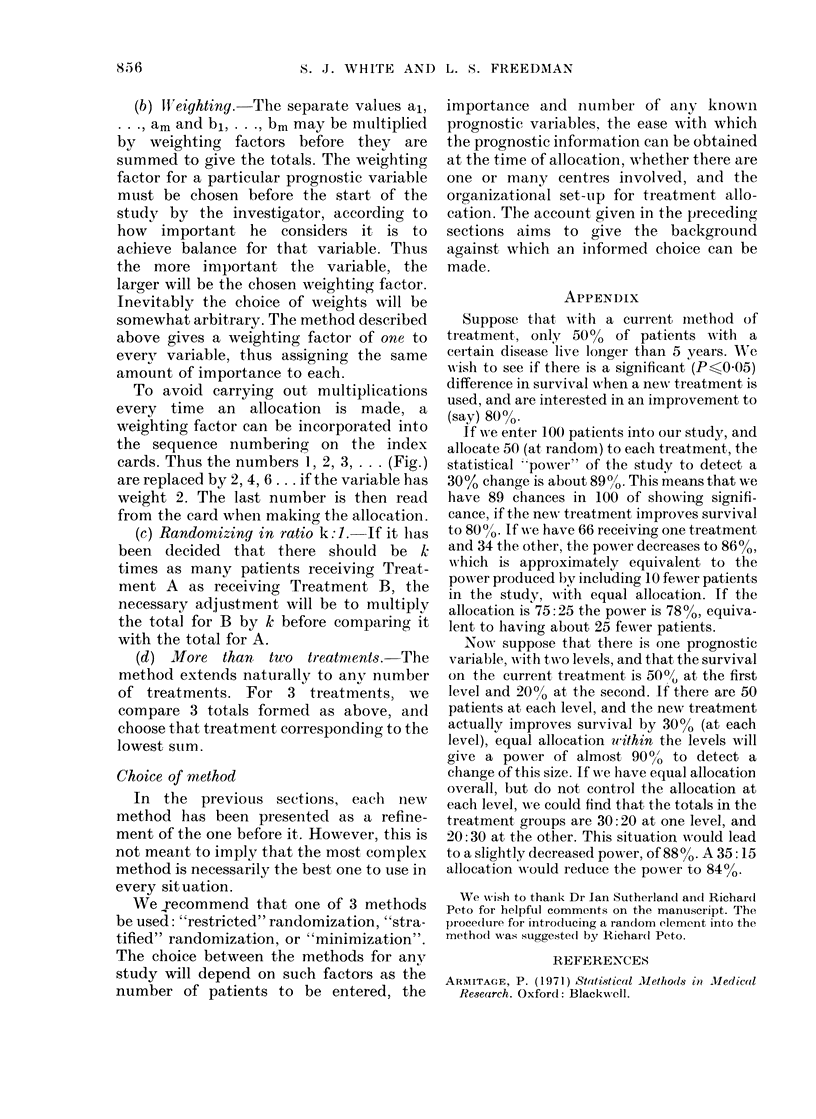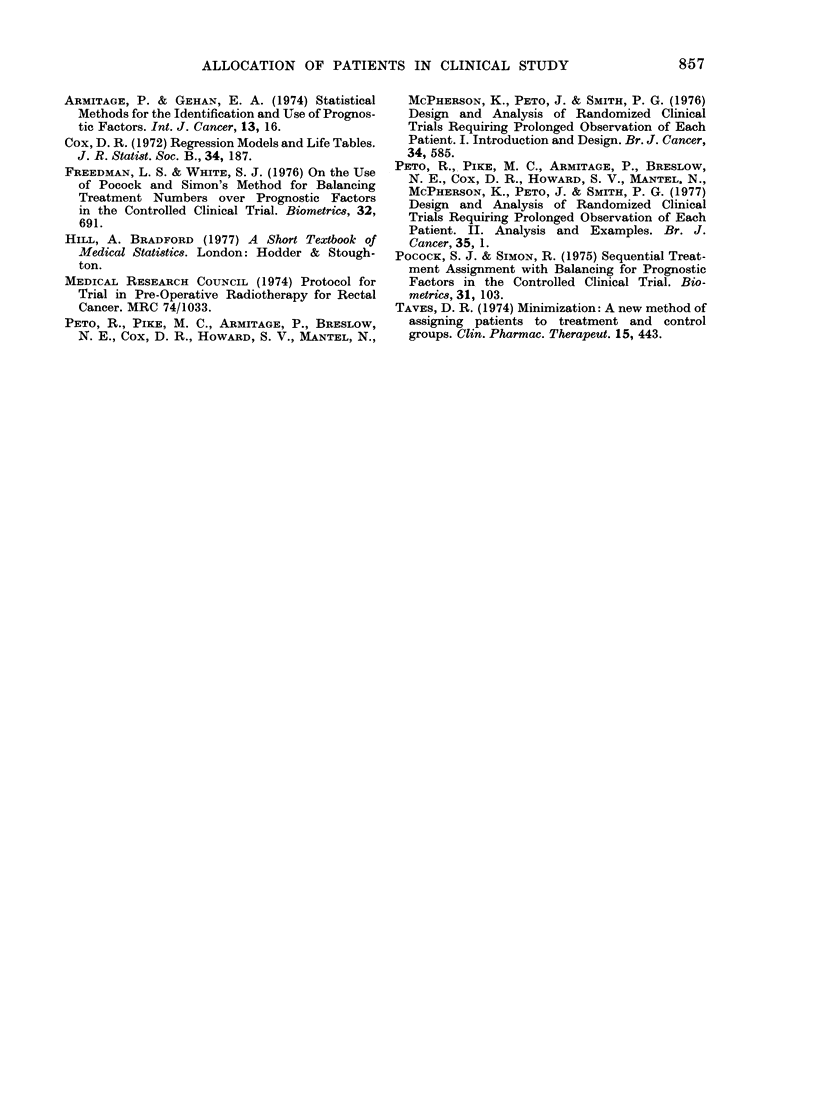# Allocation of patients to treatment groups in a controlled clinical study.

**DOI:** 10.1038/bjc.1978.124

**Published:** 1978-05

**Authors:** S. J. White, L. S. Freedman

## Abstract

In a controlled clinical study ("clinical trial") to compare 2 or more treatments for a disease, an objective method of allocating patients to treatment groups is needed. Various existing methods of allocation are described, including those which take account of patients' pre-treatment characteristics.


Br. J. Cancer (1978) **37**, 849

### ALLOCATION OF PATIENTS TO TREATMENT GROUPS IN A CONTROLLED CLINICAL STUDY

S. J. WHITE AND L. S. FREEDMAN*

*From the MRC Statistical Research and Services Unit, University College Hospital Medical School, 115 Gower Street, London WC1E 6AS*

Received 9 December 1977 Accepted 15 February 1978

**Summary.—In a controlled clinical study ("clinical trial") to compare 2 or more treatments for a disease, an objective method of allocating patients to treatment groups is needed. Various existing methods of allocation are described, including those which take account of patients' pre-treatment characteristics.**

It is often desired to compare 2 or more methods of treatment for a particular disease, where it is not known in advance which treatment is the more effective. Whether one treatment is new, or whether all are established in practice, the best comparison will be obtained by undertaking a prospective, controlled study (Hill, 1977). One question which arises early in the planning of such a study is how the patients are to be allocated to the different treatment groups, in order to obtain a meaningful comparison.

There are 2 main considerations when deciding on the method of allocation. The first is that the patients should be allocated to the treatment groups in such a way that scientifically valid conclusions can be drawn at the end of the study, giving the maximum amount of information on the relative merits of the different treatments. Statistical matters will enter into this. The second consideration is that the allocation procedure must be feasible in practice as well as in theory. In general, patients will enter the study one by one, and the treatment for each patient will need to be known soon after entry. Thus the allocation procedure must not be too complex or time-consuming. Indeed a complicated scheme could be counter-productive, in that it might deter busy clinicians from entering patients into the study.

Here we describe schemes which both provide "good" allocations, and have been found to be convenient in practice. No statistical or computational expertise is required for any of the procedures. The analysis of the results of a study is not considered here, as this has been well documented elsewhere (Armitage, 1971; Peto *et al.*, 1976, 1977).

### *Allocation schemes: the need for randomization*

The essential aim of any method of treatment allocation in a clinical study is to allow an efficient statistical comparison of the merits of the treatments to be made at the end of the investigation. We shall discuss the allocation schemes which are most reliable in achieving this end. For the most part we shall restrict our discussion to a study comparing 2 treatments, A and B (say), where approximately equal numbers of patients are to receive each treatment. Not all studies fall into this category. Some aim to compare more than two treatments (*e.g.* the Medical Research Council investigations in the treatment of rectal cancer (1974)) and in some studies it is preferable to have more patients in one treatment group than in another (Peto *et al.*, 1976). Similar considerations to those discussed here would apply in such cases, although

* Present address: MRC Cancer Trials Office, The Medical School, Hills Road, Cambridge CB2 2QH.

the methods would require some simple modifications.

We assume throughout that patients enter the study one by one, generally over some period of time.

*Subjective and deterministic procedures.*—Clinicians are used to choosing themselves the treatment which they consider is best for their patient. Such a decision will be based on their knowledge of the efficacy of the treatment, and the possibility of any unacceptable side effects. The tacit assumption behind a clinical study, however, is that it is not known initially which is the "better" of the 2 treatments for a patient. (If there are individual patients for whom one treatment is preferred, they are not included in the study.) Nevertheless it is not uncommon to find clinicians who, while having some general preference for one of the treatments, still decide to take part in an investigation. They do this because they recognise that the study will give them an evaluation of the relative merits of the 2 treatments which is more reliable than the information they already possess.

Since a clinician may have a preference for one treatment, it would not be wise to allow him to allocate the treatment by his own choice to each patient. This is because he might, for example, either consciously or unconsciously give the treatment he preferred to those patients with a better prognosis, thus hoping to ensure a good result for this treatment. Even if this is unlikely to happen, it is best to exclude the possibility completely, so that there can be no doubt in the matter at the end of the investigation.

We therefore require an allocation procedure which is not subjective, that is, not dependent on anyone's personal decision. One such method is that of "alternation". We give treatment A to the first patient who enters the study, B to the second, A to the third, and so on. This method has the advantage of being extremely simple to carry out. The very simplicity of the scheme raises a problem, however, since the clinician will be aware of the treatment which is to be allocated to the next patient. He will therefore be in a position to select the patient whom he considers most suitable for that treatment, so that the type of concealed difference mentioned above could possibly arise.

The alternation method is an example of a "deterministic" procedure. Other such procedures are allocation according to the last digit of the hospital number, first letter of surname, or month of birth. These all allow the possibility of concealed differences between the treatment groups, because the treatment allocation can be determined before the decision to enter the patient to the study has been made. In addition, there may well be other forms of bias. For example, there could be a difference in racial characteristics between the groups, if the first letter of surname is used.

*Simple and restricted randomization.*—To avoid the possibility of prediction of the next allocation, a random element is usually introduced into the procedure. The simplest form of randomization is equivalent to tossing a coin, and allocating A or B depending on whether the coin comes down "heads" or "tails". In practice a list of random numbers produced by a computer is used to give a random sequence of As and Bs (Armitage, 1971). Such a sequence is shown in Table I.

In the long run, sequences based on random numbers will lead to approximately equal numbers of As and Bs, but by chance there may sometimes be a large imbalance. From the statistical point of view it is not essential to have exactly equal numbers on the two treatments, but a more efficient comparison will generally be obtained if the numbers are as close as possible (see Appendix). Although serious losses in efficiency are unlikely to occur from a chance imbalance in numbers, it would appear sensible to rule out the possibility.

A modification of the above method is known as "restricted randomization". In this method the numbers of patients allocated to each treatment are forced to

TABLE I.—*A "Random" Allocation Sequence*

| Patient reference no. | Treatment | |
|---|---|---|
| 1 | A | |
| 2 | | B |
| 3 | | B |
| 4 | | B |
| 5 | A | |
| 6 | | B |
| 7 | A | |
| 8 | A | |
| 9 | A | |
| 10 | | B |
| 11 | A | |
| 12 | | B |
| 13 | | B |
| 14 | A | |
| 15 | | B |
| 16 | A | |
| .. | .. | .. |

be equal after every 6 patients, say, have entered the study. (The "balancing number" 6 is at the discretion of the organizer.) Thus if 3 of the first 4 patients have been allocated treatment A, the next 2 patients will necessarily be allocated B, so that after 6 patients have been entered there will be 3 in each treatment group. The same system is used for the next 6 patients, and so on, until the entry of patients is completed. Table I is, in fact, a restricted randomization list with balancing number 8.

So that it is more difficult for the investigator controlling entry of patients to predict the next allocation, he should remain unaware of the balancing number. Other devices can be introduced to prevent the pattern being detected as the study progresses. One such is to split one of the blocks of 6 (or whatever number is used) into two parts, and to insert one part at another point in the sequence.

A method of producing a restricted randomization list has been described by Peto *et al.* (1976). For a balancing number 6, each of the 20 possible sequences of 3 As and 3 Bs is associated with a different set of 2-digit numbers (*e.g.* AAABBB can be associated with the numbers 00 to 04, AABABB with 05 to 09, and so on). Pairs of digits are then read from a list of random numbers, and the corresponding sequences are written down in order.

Whatever method is used to produce the list of As and Bs, the final sequence must not, of course, be available to the investigator entering patients. If he knows in advance what the allocation for a patient will be, the purpose of the randomization is lost. One procedure is then to make out a card for each allocation. The patient serial number and allocated treatment are written on the card, and the card is sealed in an envelope bearing only the serial number. The envelopes are kept by the clinician in a pack in order of serial number, and the allocation is made by opening the next envelope in the sequence as each patient enters the study.

Another possibility is for an independent person to keep the list of As and Bs, and to tell the clinician the allocation only after he has entered a patient. This means that the procedure can be *seen* to be completely objective. A further advantage of this method in a multicentre study, where the treatment allocation is obtained by telephoning one main office, is that a central record can be kept of the patients who have been entered. It is then possible to check at a later date that no patient has been lost to the study.

*The use of prognostic information*

Before a patient starts treatment, we often have certain information which we know or suspect will be related to prognosis. For example, the patient's age, or the stage which the disease has reached before treatment starts may affect the prognosis. We shall refer to such pre-treatment items of information as "prognostic variables". In a multicentre study we may wish to see if the results vary between centres, and then "centre" is considered to be a prognostic variable.

When analysing the results of a study, we can use these variables in 3 ways. First we may see if there is any evidence that a variable does relate to prognosis. This may shed light on the disease process or on the mode of action of the treatments. It may also be valuable to the clinician in the future choice of treatment policy, and may be socially useful if it permits a more accurate prediction of the outcome for a patient. Secondly we may allow for any imbalance in the distribution of the important variables across the treatment groups, in our comparison of the treatments. Thirdly we may be able to identify subgroups of patients, in terms of the prognostic variables, who respond to treatment differently from the remainder. This last possibility requires a larger number of patients in the study than the other 2.

To achieve these aims it is desirable to have similar numbers of patients who are alike, in terms of the prognostic variables, receiving the 2 treatments. For example, if older patients might respond differently from younger patients, we would prefer to have half of our older patients receiving Treatment A, and half Treatment B. We could then see if, for older patients as distinct from younger, there was some difference in response to treatment. Similarly, if "centre" is a prognostic variable, we shall wish to have similar numbers of patients in a centre receiving each treatment.

Powerful methods of statistical analysis are available to allow for the effects of prognostic factors in the main treatment comparison, and these compensate to a large extent for an unequal treatment allocation (Armitage and Gehan, 1974; Cox, 1972). However, we shall make best use of the resources if we aim to have equal numbers for the variables which we suspect to be related to prognosis. Usually, loss of precision in the main comparison by omitting to balance for a prognostic factor will be small (see Appendix). Nevertheless, where important prognostic variables are known to exist, it would seem sensible to opt for the extra precision obtained from balanced numbers, if balance can be achieved simply. It is likely that the gain in precision in the main treatment comparison will be of most importance in studies with $<100$ patients, where several important prognostic factors exist. Balanced numbers may also be valuable when examining interactions between treatment and prognostic variables, even for larger studies.

We then wish to have an allocation scheme which takes account of each patient's values of the prognostic variables before allocating treatment. In such a scheme, it will generally only be possible to include prognostic variables which are readily available at the time the patient enters the study, since waiting for other assessments to be made could delay treatment. Such information as age, sex and diagnosis can in general be quickly obtained, but it should be remembered that details such as laboratory results may not be immediately available. It may be better not to attempt to balance for a variable, rather than have patients lost to the study because there is no time to find the necessary information.

*Stratified randomization.*—When the prognostic variables to be used in allocation have been chosen, one method which can sometimes be used is to have a separate restricted randomization list for each "stratum" defined by the variables. For example, suppose that there is one prognostic variable, age, with two "levels" (*i.e.* possible categories): over 65 and 65 or under. A separate randomization sequence is employed for each prognostic stratum, namely one sequence for the patients over 65, and one for those 65 or under (Table II).

This method of allocation is known as "stratified randomization". As with simple or restricted randomization, the procedure

TABLE II.—*"Stratified" Allocation Sequence*

| Over 65 | | | 65 or under | | |
|---|---|---|---|---|---|
| Patient no. | Treatment | | Patient no. | Treatment | |
| *E1 | A | | †Y1 | | B |
| E2 | | B | Y2 | | B |
| E3 | | B | Y3 | A | |
| E4 | A | | Y4 | A | |
| E5 | | B | Y5 | | B |
| E6 | A | | Y6 | A | |
| E7 | A | | Y7 | | B |
| E8 | | B | Y8 | A | |
| .. | .. | .. | .. | .. | .. |

* E signifies over 65

† Y signifies 65 or under.

can be implemented by using sealed envelopes or through an independent person holding the allocation sequences.

Suppose now, that a further prognostic variable, clinical stage, is to be included, having 2 levels: early stage, and late stage. The number of prognostic strata increases from 2 to 4, namely (i) over 65, early; (ii) over 65, late; (iii) 65 or under, early, and (iv) 65 or under, late. The number of separate randomization sequences is, therefore, also increased to 4.

As the number of prognostic variables increases, and especially if those variables have more than 2 levels, the total number of prognostic strata soon increases to an unmanageable size. For example, a study with 4 prognostic variables which have 2, 2, 3 and 4 levels has a total of $2 \times 2 \times 3 \times 4 = 48$ strata. If only 100 patients are to be entered, the numbers of patients in many strata will be too small for the balancing of treatment numbers, so that the method will not operate efficiently.

*A new method: minimization.*—A number of procedures have been suggested to cope with this problem, but they are often too complex to be practical. One method, sometimes known as "minimization", has been developed recently (Taves, 1974; Pocock and Simon, 1975; Freedman and White, 1976). It overcomes the difficulties of large numbers of strata by allowing the investigator to choose to balance the treatment groups in a less complete way than that attempted in stratified randomization. Stratified randomization aims to obtain equal numbers on each treatment for every possible combination of the prognostic variables, whereas the minimization method restricts its aim to equalizing treatment numbers at the different levels of each variable taken separately. In special cases where 2 prognostic variables interact, so that it is their combination which is of interest, the minimization method can be adapted to balance numbers among all combinations of that pair of variables, as well as among the remaining variables separately. Minimization has been shown to be superior to both simple and stratified randomization in producing balance for the separate prognostic variables, for the size of study most often undertaken. It is particularly superior when the number of strata is large in comparison with the number of patients (Pocock and Simon, 1975).

We shall describe a simple version of the method, and show how it can be made more sophisticated if necessary. The general method is quite complex, and often needs a small computer for its implementation, but the simplified version needs only a few hand additions of numbers.

The method aims to achieve a final distribution of treatments which is balanced with respect to each separate prognostic variable. It does this by choosing the treatment for each new patient entering the study in such a way that the "treatment imbalance" after admitting that patient is as small as possible.

For example, suppose that age is a prognostic variable, with the 2 levels "over 65" and "65 or under", and a new patient over 65 years of age is to be assigned a treatment. If there are already, for example 12 patients over 65 receiving Treatment A and 10 receiving B, the resulting imbalance if we assign this patient to A can be described by $13-10=3$. Similarly if we allocate B, the imbalance calculated in this way is $12-11=1$. If there is just this one prognostic variable, we choose to allocate B, because this gives the lesser imbalance.

If there are other prognostic variables, we calculate similar measures for each one. Then the total imbalance which would result from allocating Treatment A is described by the sum over the prognostic variables of the imbalances corresponding to A, and that for B by the sum of the imbalances corresponding to B. We then compare the 2 total values, and choose to allocate that treatment which gives the smaller treatment imbalance.

*Implementation of the method.*—Fortunately we do not need to do all the calculations detailed above at every stage, because they can be condensed to a simple procedure. Surprisingly, it turns out in practice to be easier to use as a measure of imbalance not the *difference* between the numbers in the treatment groups, were a particular treatment to be assigned, but their statistical *variance*. (In the case of 2 treatments this is equivalent to using the square of the difference.) It may not be immediately obvious that the method we describe bears any relation to using the variance measure to minimize imbalance, but it is in fact equivalent (Freedman and White, 1976). We shall describe the method for a study with a general (unspecified) number $m$ of prognostic variables.

Suppose that a new patient enters the study, and that his (or her) levels of the $m$ prognostic variables are $r_1$, $r_2$, etc., up to $r_m$. For illustration, there might be 4 prognostic variables ($m=4$) in a study of cancer therapy:

| | | |
|---|---|---|
| 1. Age | Level 1: 60 or under | Level 2: over 60 |
| 2. Sex | Level 1: male | Level 2: female |
| 3. Clinical stage | Level 1: T1 | Level 2: T2 |
| | Level 3: T3 | Level 4: T4 |
| 4. Histological grade | Level 1: well differentiated | Level 2: moderately differentiated |
| | Level 3: poorly differentiated | |

Then if the new patient is aged 58, male, stage T3 and with a poorly differentiated tumour, his levels of the 4 variables are 1, 1, 3 and 3 respectively (*i.e.* $r_1=1$, $r_2=1$, $r_3=3$ and $r_4=3$).

In order to make the allocation we look at the numbers of patients, at each of these particular levels $r_1, r_2, \ldots, r_m$ of the variables, who have been allocated A and B so far. (The numbers at the other levels are ignored.) Suppose that the numbers of patients at Level $r_1$ of Variable 1 already allocated to A and to B are $a_1$ and $b_1$ respectively. For Level $r_2$ of Variable 2, suppose that the corresponding numbers are $a_2$ and $b_2$, and so on up to Level $r_m$ of variable $m$, where the numbers already allocated to A and B are $a_m$ and $b_m$ respectively. The method is to form the 2 sums $a_1+a_2+\ldots+a_m$ and $b_1+b_2+\ldots b_m$ and to compare them to see which is the lower. We choose Treatment A for the new patient if the first sum is lower, Treatment B if the second sum is lower.

If the sums are identical, we can choose between A and B using a prepared random sequence of allocations. This sequence can be similar to that described in the section on simple randomization, with each allocation being deleted when it has been used to resolve a tie. An alternative procedure, which can be useful when a sophisticated version of the method is employed, is detailed below.

Table III shows the calculations at a particular point in a study comparing 2 treatments, where the prognostic variables and new patient are as described earlier in this section. The totals for A and B are 31 and 29 respectively, so we allocate B. It may be seen that this allocation *decreases* the imbalance for these levels of the variables age and stage, but *increases* it for sex and grade. The *total* imbalance, as defined, is however less by allocating B than by allocating A.

TABLE III.—*Computations for "Minimization" Example*

| Variable | Level | Numbers of patients already allocated to each treatment | |
|---|---|---|---|
| | | A | B |
| 1. Age | 1. 60 or under | 12 | 8 |
| 2. Sex | 1. Male | 11 | 12 |
| 3. Stage | 3. T3 | 4 | 3 |
| 4. Grade | 3. Poorly differentiated | 4 | 6 |
| | Total | 31 | 29 |

To operate the procedure conveniently, a simple way of storing information on patients already in the study is to have a

STAGE Level 3: T3

| Treatment A | | | | Treatment B | | | |
|---|---|---|---|---|---|---|---|
| 1. | 015* | 6. | | 1. | 007 | 6. | 064 |
| 2. | 036 | 7. | | 2. | 019 | 7. | |
| 3. | 050 | 8. | | 3. | 035 | 8. | |
| 4. | 061 | 9. | | 4. | 049 | 9. | |
| 5. | | 10. | | 5. | 056 | 10. | |

* Patient reference number.

FIG.—Sample index card.

set of index cards, with one card for each level of each prognostic variable (Fig.). The cards may be indexed by factor, for ease of handling. On each card we enter the study reference numbers of patients at that level of the variable, in columns according to the treatment allocated. When a new patient enters, the cards corresponding to the patient's levels of the variables are drawn from the set. The numbers $a_i$ and $b_i$ can easily be read from the cards, and the sums formed. Once the treatment is allocated, the new patient's reference number is entered on these cards in the appropriate columns, and the cards are replaced.

*Sophistications of the method: (a) random element.*—One objection which might be made is that the method is largely deterministic, and so in principle an investigator could work out in advance what a patient's treatment would be. This problem does not arise with multi-centre studies, where the information about total patient entry is only available to the central coordinator. None of the clinicians entering patients then has sufficient information to predict the next allocation.

Where a random element *is* needed, this can be introduced quite simply. We need not automatically choose the treatment which leads to the lower sum, but can instead choose according to a scheme which gives some chance of choosing the other treatment. Of course we shall still need to maintain a higher chance of choosing the treatment which leads to the lower imbalance, or the main property of the method (balance) is lost.

If there are 2 treatments, a simple way of making the procedure non-deterministic is to add a (positive or negative) "random" number to the total for A, before comparing it with the total for B. An appropriate list of random numbers is prepared at the beginning of the study, and each number is deleted as it is used. For instance, the list could consist of the numbers $-4$, $-3$, $-2$, $-1$, 0, 1, 2, 3, 4, each occurring equally often in a random fashion. If for example the total for A is 2 more than the total for B, adding the random number will lead to B being chosen 6/9 of the time (when $-1$, 0, 1, 2, 3, or 4 appear), A being chosen 2/9 of the time (when $-4$ or $-3$ appear), and a tie occurring 1 time in 9 (when $-2$ appears).

The possibility of a tie can be avoided altogether when this method is used, as the values $+\frac{1}{2}$ and $-\frac{1}{2}$ can also be added to the total for A (each used equally often, at random). Thus the random list will consist of the numbers $-4\frac{1}{2}$, $-3\frac{1}{2}$, $-2\frac{1}{2}$, $-1\frac{1}{2}$, $-\frac{1}{2}$, $\frac{1}{2}$, $1\frac{1}{2}$, $2\frac{1}{2}$, $3\frac{1}{2}$ and $4\frac{1}{2}$. In the example above, B will then be chosen with probability 7/10, and A with probability 3/10. If the total for A is 4 more than the total for B, B will be chosen with probability 9/10, and A with probability only 1/10. Thus as the potential imbalance becomes larger, the probability of choosing the treatment leading to the lower imbalance also increases. This preserves the good balancing properties of the scheme.

Use of the numbers illustrated above will give a deterministic allocation whenever the difference between the totals is 5 or more. This is because addition of the random number to one total can make no difference to which total is the lower. A way of avoiding this is to replace the values $-4\frac{1}{2}$ and $4\frac{1}{2}$ by $-1000$ and 1000 respectively. When the difference between the totals is 4 or more, there will then be always a 1/10 chance of *not* choosing the treatment leading to the smaller imbalance.

The introduction of the random element has certain disadvantages, in that the procedure then takes longer to carry out, and there is a greater possibility of imbalance creeping in. Its introduction is often not necessary.

(*b*) *Weighting*.—The separate values $a_1, \ldots, a_m$ and $b_1, \ldots, b_m$ may be multiplied by weighting factors before they are summed to give the totals. The weighting factor for a particular prognostic variable must be chosen before the start of the study by the investigator, according to how important he considers it is to achieve balance for that variable. Thus the more important the variable, the larger will be the chosen weighting factor. Inevitably the choice of weights will be somewhat arbitrary. The method described above gives a weighting factor of *one* to every variable, thus assigning the same amount of importance to each.

To avoid carrying out multiplications every time an allocation is made, a weighting factor can be incorporated into the sequence numbering on the index cards. Thus the numbers 1, 2, 3, . . . (Fig.) are replaced by 2, 4, 6 . . . if the variable has weight 2. The last number is then read from the card when making the allocation.

(*c*) *Randomizing in ratio* k*:1*.—If it has been decided that there should be $k$ times as many patients receiving Treatment A as receiving Treatment B, the necessary adjustment will be to multiply the total for B by $k$ before comparing it with the total for A.

(*d*) *More than two treatments*.—The method extends naturally to any number of treatments. For 3 treatments, we compare 3 totals formed as above, and choose that treatment corresponding to the lowest sum.

*Choice of method*

In the previous sections, each new method has been presented as a refinement of the one before it. However, this is not meant to imply that the most complex method is necessarily the best one to use in every situation.

We recommend that one of 3 methods be used: "restricted" randomization, "stratified" randomization, or "minimization". The choice between the methods for any study will depend on such factors as the number of patients to be entered, the importance and number of any known prognostic variables, the ease with which the prognostic information can be obtained at the time of allocation, whether there are one or many centres involved, and the organizational set-up for treatment allocation. The account given in the preceding sections aims to give the background against which an informed choice can be made.

### Appendix

Suppose that with a current method of treatment, only 50% of patients with a certain disease live longer than 5 years. We wish to see if there is a significant ($P \leqslant 0{\cdot}05$) difference in survival when a new treatment is used, and are interested in an improvement to (say) 80%.

If we enter 100 patients into our study, and allocate 50 (at random) to each treatment, the statistical "power" of the study to detect a 30% change is about 89%. This means that we have 89 chances in 100 of showing significance, if the new treatment improves survival to 80%. If we have 66 receiving one treatment and 34 the other, the power decreases to 86%, which is approximately equivalent to the power produced by including 10 fewer patients in the study, with equal allocation. If the allocation is 75:25 the power is 78%, equivalent to having about 25 fewer patients.

Now suppose that there is one prognostic variable, with two levels, and that the survival on the current treatment is 50% at the first level and 20% at the second. If there are 50 patients at each level, and the new treatment actually improves survival by 30% (at each level), equal allocation *within* the levels will give a power of almost 90% to detect a change of this size. If we have equal allocation overall, but do not control the allocation at each level, we could find that the totals in the treatment groups are 30:20 at one level, and 20:30 at the other. This situation would lead to a slightly decreased power, of 88%. A 35:15 allocation would reduce the power to 84%.

We wish to thank Dr Ian Sutherland and Richard Peto for helpful comments on the manuscript. The procedure for introducing a random element into the method was suggested by Richard Peto.

### REFERENCES

Armitage, P. (1971) *Statistical Methods in Medical Research*. Oxford: Blackwell.

Armitage, P. & Gehan, E. A. (1974) Statistical Methods for the Identification and Use of Prognostic Factors. *Int. J. Cancer*, **13**, 16.

Cox, D. R. (1972) Regression Models and Life Tables. *J. R. Statist. Soc.* B., **34**, 187.

Freedman, L. S. & White, S. J. (1976) On the Use of Pocock and Simon's Method for Balancing Treatment Numbers over Prognostic Factors in the Controlled Clinical Trial. *Biometrics*, **32**, 691.

Hill, A. Bradford (1977) *A Short Textbook of Medical Statistics*. London: Hodder & Stoughton.

Medical Research Council (1974) Protocol for Trial in Pre-Operative Radiotherapy for Rectal Cancer. MRC 74/1033.

Peto, R., Pike, M. C., Armitage, P., Breslow, N. E., Cox, D. R., Howard, S. V., Mantel, N., McPherson, K., Peto, J. & Smith, P. G. (1976) Design and Analysis of Randomized Clinical Trials Requiring Prolonged Observation of Each Patient. I. Introduction and Design. *Br. J. Cancer*, **34**, 585.

Peto, R., Pike, M. C., Armitage, P., Breslow, N. E., Cox, D. R., Howard, S. V., Mantel, N., McPherson, K., Peto, J. & Smith, P. G. (1977) Design and Analysis of Randomized Clinical Trials Requiring Prolonged Observation of Each Patient. II. Analysis and Examples. *Br. J. Cancer*, **35**, 1.

Pocock, S. J. & Simon, R. (1975) Sequential Treatment Assignment with Balancing for Prognostic Factors in the Controlled Clinical Trial. *Biometrics*, **31**, 103.

Taves, D. R. (1974) Minimization: A new method of assigning patients to treatment and control groups. *Clin. Pharmac. Therapeut.* **15**, 443.